# Supplementary material for: Cost-Effectiveness of a School-and Family-Based Childhood Obesity Prevention Programme in China: The “CHIRPY DRAGON” Cluster-Randomised Controlled Trial
Source: Int J Public Health. 2021 Aug 25;66:1604025. doi: 10.3389/ijph.2021.1604025 (PMC8439195; doi:10.3389/ijph.2021.1604025)
Supplement: Supplementary file 2 [file DataSheet1.docx]

**Table A** CHEERS Checklist - Items to include when reporting economic evaluations of health interventions

| **Section/item** | **Item No** | **Recommendation** | **Reported on page No** |
| --- | --- | --- | --- |
| **Title and abstract** | | | |
| Title | 1 | Identify the study as an economic evaluation or use more specific terms such as “cost-effectiveness analysis”, and describe the interventions compared. | Page 1 |
| Abstract | 2 | Provide a structured summary of objectives, perspective, setting, methods (including study design and inputs), results (including base case and uncertainty analyses), and conclusions. | Page 1 |
| **Introduction** | | | |
| Background and objectives | 3 | Provide an explicit statement of the broader context for the study. | Pages 1-2 |
|  |  | Present the study question and its relevance for health policy or practice decisions. | Page 2 |
| **Methods** | | | |
| Target population and subgroups | 4 | Describe characteristics of the base case population and subgroups analysed, including why they were chosen. | Page 2 |
| Setting and location | 5 | State relevant aspects of the system(s) in which the decision(s) need(s) to be made. | Page 2 |
| Study perspective | 6 | Describe the perspective of the study and relate this to the costs being evaluated. | Pages 2-3 |
| Comparators | 7 | Describe the interventions or strategies being compared and state why they were chosen. | Page 2 |
| Time horizon | 8 | State the time horizon(s) over which costs and consequences are being evaluated and say why appropriate. | Page 2 |
| Discount rate | 9 | Report the choice of discount rate(s) used for costs and outcomes and say why appropriate. | Page 4 |
| Choice of health outcomes | 10 | Describe what outcomes were used as the measure(s) of benefit in the evaluation and their relevance for the type of analysis performed. | Page 3 |
| Measurement of effectiveness | 11a | *Single study-based estimates:*Describe fully the design features of the single effectiveness study and why the single study was a sufficient source of clinical effectiveness data. | Pages 2-3 |
|  | 11b | *Synthesis-based estimates*: Describe fully the methods used for identification of included studies and synthesis of clinical effectiveness data. | Pages 2-3 |
| Measurement and valuation of preference based outcomes | 12 | If applicable, describe the population and methods used to elicit preferences for outcomes. | Page 3 |
| Estimating resources and costs | 13a | *Single study-based economic evaluation:* Describe approaches used to estimate resource use associated with the alternative interventions. Describe primary or secondary research methods for valuing each resource item in terms of its unit cost. Describe any adjustments made to approximate to opportunity costs. | Pages 2-3 |
|  | 13b | *Model-based economic evaluation:*Describe approaches and data sources used to estimate resource use associated with model health states. Describe primary or secondary research methods for valuing each resource item in terms of its unit cost. Describe any adjustments made to approximate to opportunity costs. | N/A |
| Currency, price date, and conversion | 14 | Report the dates of the estimated resource quantities and unit costs. Describe methods for adjusting estimated unit costs to the year of reported costs if necessary. Describe methods for converting costs into a common currency base and the exchange rate. | Page 3 |
| Choice of model | 15 | Describe and give reasons for the specific type of decision-analytical model used. Providing a figure to show model structure is strongly recommended. | N/A |
| Assumptions | 16 | Describe all structural or other assumptions underpinning the decision-analytical model. | N/A |
| Analytical methods | 17 | Describe all analytical methods supporting the evaluation. This could include methods for dealing with skewed, missing, or censored data; extrapolation methods; methods for pooling data; approaches to validate or make adjustments (such as half cycle corrections) to a model; and methods for handling population heterogeneity and uncertainty. | Page 4 |
| **Results** | | | |
| Study parameters | 18 | Report the values, ranges, references, and, if used, probability distributions for all parameters. Report reasons or sources for distributions used to represent uncertainty where appropriate. Providing a table to show the input values is strongly recommended. | N/A |
| Incremental costs and outcomes | 19 | For each intervention, report mean values for the main categories of estimated costs and outcomes of interest, as well as mean differences between the comparator groups. If applicable, report incremental cost-effectiveness ratios. | Pages 5-6 |
| Characterising uncertainty | 20a | *Single study-based economic evaluation:* Describe the effects of sampling uncertainty for the estimated incremental cost and incremental effectiveness parameters, together with the impact of methodological assumptions (such as discount rate, study perspective). | Pages 5-6 |
|  | 20b | *Model-based economic evaluation:*Describe the effects on the results of uncertainty for all input parameters, and uncertainty related to the structure of the model and assumptions. | N/A |
| Characterising heterogeneity | 21 | If applicable, report differences in costs, outcomes, or cost-effectiveness that can be explained by variations between subgroups of patients with different baseline characteristics or other observed variability in effects that are not reducible by more information. | N/A |
| **Discussion** | | | |
| Study findings, limitations, generalisability, and current knowledge | 22 | Summarise key study findings and describe how they support the conclusions reached. Discuss limitations and the generalisability of the findings and how the findings fit with current knowledge. | Pages 6-7 |
| **Other** | | | |
| Source of funding | 23 | Describe how the study was funded and the role of the funder in the identification, design, conduct, and reporting of the analysis. Describe other non-monetary sources of support. | Page 8 |
| Conflicts of interest | 24 | Describe any potential for conflict of interest of study contributors in accordance with journal policy. In the absence of a journal policy, we recommend authors comply with International Committee of Medical Journal Editors recommendations. | Page 8 |

For consistency, the CHEERS Statement checklist format is based on the format of the CONSORT statement checklist

**Table B** Resource use and associated unit costs (Yuan, 2016/2017 Prices) for public sector perspective

| Type | Resource use item for intervention delivery | Unit cost (Yuan) | Quantity  for 12 months | Total | Source |
| --- | --- | --- | --- | --- | --- |
| Office stationery | Ink pads (Components 1, 2, 3 and 4) | 4.9/each | 4 | 19.6 | Online (taobao.com) |
|  | Permanent markers (Components 1, 2, 3 and 4) | 1.2/each | 12 | 14.4 | Online (taobao.com) |
| Printing | Colourful educational leaflets for parents and families (Component 1) | 0.58/each | 1950 | 1131 | Feida Tu Wen store |
|  | Family healthy behaviour challenges fun cards  (Components 1 and 3) | 0.15/each | 27000 | 4050 | Feida Tu Wen store |
|  | Stickers (with CHIRPY DRAGON logo)  (Components 1 and 3) | 0.14/each | 3700 | 500 | Caiyi Bangong Haocai store |
|  | Record cards of individual performance  (Components 1 and 3) | 6.5/each | 111 | 721.5 | Wanmei Chongyin store |
|  | Illustrative photo cards for child workshops  (first semester) (Component 1) | 0.4/each card | 2030 | 819 | Wanmei Chongyin store |
|  | Illustrative photo cards for child workshops  (second semester) (Component 1) | 0.4/each card | 1190 | 476 | Wanmei Chongyin store |
|  | Illustrative photo cards for child carers workshops  (Component 1) | 0.35/each card | 7300 | 2580 | Wanmei Chongyin store |
|  | Teaching boards for child carers workshops  (Shahe School) (Component 1) | 0.15/each note | 40 | 6 | Wanmei Chongyin store |
|  | Family healthy behaviour challenges reward board  (Components 1 and 3) | 0.29/each | 3000 | 850 | Feida Tu Wen store |
| Labour | Workshops assistant (each practice = 2 hours)  (Component 3) | 30.5/each practice | 77 | 2348 | Standard monthly salary rate (Guangzhou CDC) |
|  | Hour of CHIRPY DRAGON teachers’ time  (Components 1, 2, 3 and 4) | 50/hr | 708.5 | 35425 | Standard rates provided by Guangzhou CDC |
| Delivery fee | Family healthy behaviour challenges reward board  (Components 1 and 3) | 20/delivery | 2 | 40 | Guangjun Tourism |
|  | Loudspeakers (Components 1 and 3) | 12/delivery | 2 | 24 | Guangzhou transportation group taxi Co. Ltd |
| **Table B** Resource use and unit costs (Yuan, 2016/17 Prices), public sector perspective (continued) | | | | | |
| Type | Resource use item for intervention delivery | Unit cost (Yuan) | Quantity  for 12 months  for 12 month | Total annual cost | Source |
| Workshops materials | PowerPoint remote control (Component 1) | 32/each | 7 | 224 | Online (taobao.com) |
|  | Canister (Component 1) | 1/each | 30 | 30 | Online (taobao.com) |
|  | Measuring spoon (Component 1) | 6/each | 3 | 18 | Online (taobao.com) |
|  | AA batteries (Component 1) | 3.1/each | 25 | 77 | Jinli store |
|  | Stamp (Components 1 and 3) | 30/each | 3 | 90 | Jinli store |
|  | Transparent plastic bag (Component 1) | 0.01/each | 200 | 19.4 | Online (taobao.com) |
|  | Suitcase (Components 1 and 3) | 99/each | 2 | 198 | Online (taobao.com) |
|  | Cart (Component 1 and 3) | 36.8/each | 3 | 110.4 | Online (taobao.com) |
|  | Paper plates for child workshops (Component 1) | 0.19/each plate | 1500 | 295 | Online (taobao.com) |
|  | Electronic scale (Component 1) | 23/each | 3 | 69 | Online (taobao.com) |
|  | Food used in child carer workshops (Component 1) | 19.8/each workshop | 40 | 793 | Local market, Wal-Mart |
|  | Canister (larger) (Component 1) | 2.42/each | 6 | 14.5 | Online (taobao.com) |
|  | Loudspeakers (Components 1 and 3) | 160/each | 5 | 800 | Online (taobao.com) |
|  | Stamp (with CHIRPY DRAGON logo)  (Components 1 and 3) | 22.5/each | 10 | 225 | Qingqing Wenxue store |
|  | Balloon (Component 3) | 0.1/each | 2000 | 200 | Online (taobao.com) |
|  | Finger board used in physically active family friendly games (Component 3) | 37.5/each | 2 | 75 | Online (taobao.com) |
|  | Pencils (Components 1 and 3) | 6/each | 5 | 30 | Online (taobao.com) |
|  | Rubber band (Component 3) | 0.008/each | 2000 | 15 | Online (taobao.com) |
|  | Megaphone (Components 1 and 3) | 40/each | 6 | 240 | Online (taobao.com) |
|  | Strips of cloth (Component 3) | 1/each | 100 | 100 | Qingqing Wenxue store |
| Incentives | Incentive prizes for meeting family healthy behaviour challenges (Components 1 and 3) | 9.7/each | 991 | 9620 | Jinli store,  Qingqing Wenxue store |
|  | Recognition certificates for catering teams  (Component 2) | 14.8/each | 33 | 489 | Online (taobao.com) |
| Teachers  telephone | CHIRPY DRAGON teachers’ average cost of mobile phone (Components 1, 2, 3 and 4) | 269.8/month | 10 | 2698 | China mobile |
| Teachers  transport | CHIRPY DRAGON teachers’ average cost of transport (Components 1, 3 and 4)  (Components 1, 3 and 4) | 744.25/month | 12 | 8931 | China National Railways |

**Table C** Mean resource use and costs for each intervention component (by class and child)

| Intervention component | Resource type | Mean annual resource use per class | Mean annual cost per class, Yuan | Average cost per child  (assuming average class size of 45),  Yuan |
| --- | --- | --- | --- | --- |
| Component 1:  Improving childhood obesity related knowledge and behaviour among children and their main carers | Labour: CHIRPY DRAGON teachers time (hours)  Interactive educational activities for main carers *****  Interactive educational activities for children  Quiz for main cares and children *****  Family-wide healthy behavioural challenges  Office stationery  Ink pads  Permanent markers  Printing  Colourful educational leaflets for parents and families *****  Family healthy behaviour challenges fun cards  Stickers (with CHIRPY DRAGON logo)  Record cards of individual performance  Illustrative photo cards for child workshops (first semester)  Illustrative photo cards for child workshops (second semester)  Illustrative photo cards for child carers workshops *****  Teaching boards for child carers workshops (Shahe School) *****  Family healthy behaviour challenges reward board  Delivery fee  Family healthy behaviour challenges reward board  Loudspeakers *****  Workshops materials  PowerPoint remote control  Canister  Measuring spoon  AA batteries  Stamp  Transparent plastic bag  Suitcase  Cart  Paper plates for child workshops  Electronic scale  Canister (larger)  Loudspeakers *****  Stamp (with CHIRPY DRAGON logo)  Pencils  Megaphone  Food presenting in child carers workshops  Incentives: Family healthy behavioural challenges  Incentive prize  Telephone  CHIRPY DRAGON teacher telephone  Transport  CHIRPY DRAGON teacher transport | 4.85  2.32  0.9  2.56  0.01  0.04  97.5  238.12  32.64  0.98  23.88  14  365  2  26.47  0.017  0.08  0.08  0.35  0.04  0.29  0.03  2.35  0.02  0.03  17.64  0.04  0.07  0.2  0.09  0.04  0.06  0.56  8.24  0.03  0.06 | 242.5  115.88  45  128.38  0.06  0.04  56.55  35.73  4.41  6.37  9.64  5.6  129  0.3  7.50  0.21  0.96  2.64  0.35  0.21  0.91  0.79  0.23  1.86  1.04  3.47  0.81  0.17  32  1.99  0.26  2.26  9.35  95.32  7.93  46.70 | 5.38  2.58  1  2.85  0.001  0.0008  1.25  0.79  0.10  0.14  0.21  0.12  2.87  0.007  0.17  0.005  0.021  0.06  0.008  0.005  0.02  0.017  0.005  0.04  0.02  0.08  0.02  0.004  0.71  0.04  0.006  0.05  0.20  2.12  0.18  1.04 |
| Component 1 total mean intervention running/delivery cost per school  Component 1 total mean intervention running/delivery cost per class  Component 1 total mean intervention running/delivery cost per child assuming a class of 45  Component 1 total mean intervention running/delivery cost per consented child (on average 41 per class) | | | 2549.52 Yuan (718.17 $, 504.85 £)  996.42 Yuan (280.68 $, 197.33 £)  22.12 Yuan (6.23 $, 4.38 £)  24.30 Yuan (6.84 $, 4.81 £) | |
| Component 2:  Improving the nutritional quality of school lunch provision | Labour: CHIRPY DRAGON teachers time (hours)  Supportive regular evaluations and feedbacks to the catering teams  Office stationery  Ink pads  Permanent markers  Incentives: Catering teams  Recognition certificate  Telephone  CHIRPY DRAGON teacher telephone | 0.01  0.01  0.04  0.51  0.03 | 0.59  0.06  0.04  5.75  7.93 | 0.01  0.001  0.0008  0.13  0.18 |
| Component 2 total mean intervention running/delivery cost per school  Component 2 total mean intervention running/delivery cost per class  Component 2 total mean intervention running/delivery cost per child assuming a class of 45  Component 2 total mean intervention running/delivery cost per consented child (on average 41 per class) | | | 61.1 Yuan (17.21 $, 12.09 £)  14.37 Yuan (4.05 $, 2.85 £)  0.32 Yuan (0.09 $, 0.06 £)  0.35 Yuan (0.10 $, 0.07 £) | |
| Component 3:  Increasing children’s physical activity level outside school | Labour: CHIRPY DRAGON teachers time (hours)  Physically active family friendly games learnt and practiced at school for children and their parents *****  Family-wide healthy behavioural challenges  Workshops assistant time (hours) *****  Office stationery  Ink pads  Permanent markers  Printing  Family healthy behaviour challenges fun cards  Stickers (with CHIRPY DRAGON logo)  Record cards of individual performance  Family healthy behaviour challenges reward board  Delivery fee  Family healthy behaviour challenges reward board  Loudspeakers *****  Workshops materials  Stamp *****  Suitcase *****  Cart *****  Loudspeakers *****  Stamp (with CHIRPY DRAGON logo) *****  Balloon *****  Finger board used in physically active family friendly games *****  Pencils *****  Rubber band *****  Megaphone *****  Strips of cloth *****  Incentives: Family healthy behavioural challenges  Incentive prize  Telephone  CHIRPY DRAGON teacher telephone  Transport  CHIRPY DRAGON teacher transport ***** | 4.52  0.86  3.85  0.01  0.04  79.41  10.88  0.33  8.82  0.006  0.02  0.04  0.02  0.03  0.05  0.12  100  0.1  0.06  100  0.06  5  3.41  0.03  0.06 | 226.25  42.8  117.4  0.06  0.04  11.91  1.47  2.12  2.49  0.07  0.24  1.12  1.98  1.1  8  2.81  10  3.75  0.37  0.75  2.4  5  15.71  7.93  49.61 | 5.03  0.95  2.61  0.001  0.0008  0.26  0.03  0.05  0.06  0.002  0.005  0.02  0.04  0.02  0.18  0.06  0.22  0.08  0.008  0.02  0.053  0.11  0.35  0.18  1.10 |
| Component 3 total mean intervention running/delivery cost per school  Component 3 total mean intervention running/delivery cost per class  Component 3 total mean intervention running/delivery cost per child assuming a class of 45  Component 3 total mean intervention running/delivery cost per consented child (on average 41 per class) | | | 790.41 Yuan (222.65 $, 156.51 £)  515.38 Yuan (145.18 $, 102.05 £)  11.43 Yuan (3.22 $, 2.26 £)  12.57 Yuan (3.54 $, 2.49 £) | |
| Component 4:  Increasing children’s physical activity level in school | Labour: CHIRPY DRAGON teachers time (hours)  Monthly meeting with relevant school staff and student representatives  Office stationery  Ink pads  Permanent markers  Telephone  CHIRPY DRAGON teacher telephone  Transport  CHIRPY DRAGON teacher transport | 0.16  0.01  0.04  0.03  0.08 | 8.23  0.06  0.04  7.93  58.37 | 0.18  0.001  0.0008  0.18  1.30 |
| Component 4 total mean intervention running/delivery cost per school  Component 4 total mean intervention running/delivery cost per class  Component 4 total mean intervention running/delivery cost per child assuming a class of 45  Component 4 total mean intervention running/delivery cost per consented child (on average 41 per class) | | | 317.23 Yuan (89.36 $, 62.81 £)  74.63 Yuan (21.02 $, 14.77 £)  1.66 Yuan (0.47 $, 0.33 £)  1.82 Yuan (0.51 $, 0.36 £) | |
| Total mean intervention running/delivery cost per school  Total mean intervention running/delivery cost per class  Total mean intervention running/delivery cost per child assuming a class of 45  Total mean intervention running/delivery cost per consented child (averagely 41 per class) | | | 3718.26 Yuan (1047.39 $, 736.28 £)  1600.8 Yuan (449.73 $, 317 £)  35.53 Yuan (10.01 $, 7.04 £)   - 1. Yuan (10.97 $, 7.73 £) | |

Notes: Total number of intervention schools (n = 20), Total number of intervention classes (n = 85), Total number of intervention consented classes (20), Assumed average class size (45), Total number of intervention consented children (n = 832). Mean cost per class: the total cost for delivery across the whole intervention arm for the specific resource type divided by the number of classes (either 85 or 20)**.** Some parts of the intervention, which involved family members, were only delivered to intervention consented classes (1 class per school). These costs were collected at class level and were related to interactive educational activities for main carers, quiz for main carers and children; and physically active family friendly games learnt and practiced at school for children and their parents. These resource type costs are indicated with ***** in the table. The rest of the intervention was delivered to all year one children (85 classes in 20 schools).

**Table D** Resource use and cost associated with development of intervention (internal/pre implementation preparation)

| Component | Resource type | Total  resource use | Total  Cost for 20 intervention schools, Yuan |
| --- | --- | --- | --- |
| Development of schoolteacher handbook  (Explanation of the intervention) | Research staff time (hours)  Research Fellow  Senior Research Fellow | 6  2 | 402  144 |
| Hiring of a designer  (To optimise the presentation of developed intervention materials (leaflets and illustration media)) | Designer time (month) | 2 | 20000 |
| Researcher preparation time  (For CHIRPY DRAGON teachers training) | Research staff time (hours)  Research Associate  Research Fellow | 12  6 | 744  402 |
| Staff meeting at each school  (To discuss their current situation about children’s physical activity) | CHIRPY DRAGON teachers time (hours)  CHIRPY DRAGON travel costs (transport)  Return train ticket | 10  20 | 500  80 |
|  | | | 22272 Yuan  (6273 $, 4410 £) |

Notes: Total number of intervention schools (n = 20), Total number of intervention classes (n = 85), Total number of intervention consented classes (20), Assumed average class size (45), Total number of intervention consented children (n = 832)

**Table E** Mean resource use and cost associated with initial implementation of intervention

| Component | Resource type | Mean annual resource use per class | Mean annual cost per class, Yuan | Average cost per child  (assuming average class size of 45),  Yuan |
| --- | --- | --- | --- | --- |
| Workshops/sessions training  (CHIRPY DRAGON teachers training to deliver workshops/sessions) | Research staff time (hours)  Research Associate  Research Fellow  Research associate and research fellow travel costs  Return train ticket  CHIRPY DRAGON teachers time (hours) | 0.32  0.17  0.16  1.62 | 18.69  11.98  0.65  81.17 | 0.42  0.27  0.01  1.80 |
| Initial printing of handbooks | Number of handbooks | 0.47 | 0.18 | 0.004 |
| Intervention set-up meeting  (To explain about the intervention components to school staff) | CHIRPY DRAGON teachers time (hours)  School principal travel costs (transport)  Return train ticket | 0.02  0.24 | 1.18  0.94 | 0.03  0.02 |
| Catering team introduction meeting  (Managers and chefs meeting for introducing five school lunch improvement objectives) | CHIRPY DRAGON teachers time (hours)  Catering team time (hours)  Managers and chefs’ time  Managers and chefs travel costs (transport)  Return train ticket | 0.01  0.42  0.42 | 0.59  31.34  1.69 | 0.01  0.69  0.04 |
| Total mean intervention implementation cost per school  Total mean intervention implementation cost per class    Total mean intervention implementation cost per child assuming a class of 45  Total mean intervention implementation cost per consented child (averagely 41 per class) | | | 630.74 Yuan (177.67 $, 124.89 £)  148.41 Yuan (41.80 $, 29.39 £)  3.29 Yuan (0.92 $, 0.65 £)  3.62 Yuan (1.02 $, 0.71 £) | |

Notes: Total number of intervention schools (n = 20), Total number of intervention classes (n = 85), Total number of intervention consented classes (20), Assumed average class size (45), Total number of intervention consented children (n = 832)

**Table F -** Mean (SD) cost of lunch per diner per day and per year in each schoo**l**

|  | School | Mean (SD) cost of lunch per school per day over the 12 month trial period in Yuan | Mean (SD) number of diners per school per day over the 12 month trial period | Mean (SD) cost of lunch per diner per day in Yuan * | Average cost of lunch per diner per year in Yuan ** | Average cost of lunch per diner per year in US$ | Average cost of lunch per diner per year in £ |
| --- | --- | --- | --- | --- | --- | --- | --- |
| Intervention  group | 1 | 3740.49 (218.47) | 596 (0) | 6.28 (0.36) | 1570 | 442.25 | 310.89 |
|  | 2 | 3419.88 (330.16) | 465.95 (37.91) | 7.33 (0.26) | 1832.5 | 516.19 | 362.87 |
|  | 3 | 4149.05 (142.41) | 643.76 (2.92) | 6.44 (0.21) | 1610 | 453.52 | 318.81 |
|  | 4 | 3667.24 (2641.25) | 362.78 (202.49) | 8.24 (3.38) | 2060 | 580.28 | 407.92 |
|  | 5 | 4807.07 (144.92) | 892.92 (10.58) | 5.38 (0.16) | 1345 | 378.87 | 266.34 |
|  | 6 | 1494.82 (97.28) | 265.93 (5.58) | 5.62 (0.33) | 1405 | 395.77 | 278.21 |
|  | 7 | 4149.42 (178.72) | 756.75 (13.45) | 5.48 (0.21) | 1370 | 385.91 | 271.28 |
|  | 8 | 7077.36 (143.39) | 1040.79 (20.54) | 6.8 (0.03) | 1700 | 478.87 | 336.63 |
|  | 9 | 4620.75 (1387.71) | 568.78 (163.84) | 8.09 (0.39) | 2022.5 | 569.72 | 400.49 |
|  | 10 | 8553.28 (44.18) | 1006.26 (5.19) | 8.5 (0) | 2125 | 598.59 | 420.79 |
|  | 11 | 7834.54 (5640.71) | 784.94 (55.14) | 9.38 (0.44) | 2345 | 660.56 | 464.35 |
|  | 12 | 4622.03 (847.66) | 477.97 (23.38) | 9.7 (1.8) | 2425 | 683.09 | 480.19 |
|  | 13 | 1996.13 (1446.23) | 275.73 (260.63) | 8.22 (1.27) | 2055 | 578.87 | 406.93 |
|  | 14 | 4296.01 (557.45) | 641.96 (73.29) | 6.69 (0.37) | 1672.5 | 471.12 | 331.18 |
|  | 15 | 828.07 (28.16) | 148.01 (3.93) | 5.59 (0.07) | 1397.5 | 393.66 | 276.73 |
|  | 16 | 4691.11 (546.03) | 873.51 (71.59) | 5.38 (0.5) | 1345 | 378.87 | 266.34 |
|  | 17 | 4625.72 (613.50) | 597.59 (7.22) | 7.88 (0.23) | 1970 | 554.92 | 390.09 |
|  | 18 | 4105.75 (277.65) | 668.98 (42.80) | 6.13 (0.18) | 1532.5 | 431.69 | 303.46 |
| Total  Mean (SD) |  | 4469.12 (2433.28) | 631.24 (257.88) | 7.06 (1.66) | 1765 | 497.18 | 349.50 |
|  | School | Mean (SD) cost of lunch per school per day over the 12 month trial period in Yuan | Mean (SD) number of diners per school per day over the 12 month trial period | Mean (SD) cost of lunch per diner per day in Yuan * | Average cost of lunch per diner per year in Yuan ** | Average cost of lunch per diner per year in US$ | Average cost of lunch per diner per year in £ |
| Control  group | 1 | 2763.93 (0.17) | 500 (0) | 5.53 (0) | 1382.5 | 389.44 | 273.76 |
|  | 2 | 757.42 (179.26) | 110.159 (18.18) | 6.94 (1.56) | 1735 | 488.73 | 343.56 |
|  | 3 | 331.66 (0.23) | 60 (0) | 5.52 (0) | 1380 | 388.73 | 273.27 |
|  | 4 | 2159.56 (25.65) | 398.2 (3.88) | 5.42 (0.02) | 1355 | 381.69 | 268.32 |
|  | 5 | 9355.67 (1147.67) | 1325.12 (34.47) | 7.05 (0.79) | 1762.5 | 496.48 | 349.01 |
|  | 6 | 7579.64 (426.62) | 1363.23 (48.39) | 5.56 (0.38) | 1390 | 391.54 | 275.25 |
|  | 7 | 3815.64 (680.06) | 543.85 (25.84) | 7.02 (1) | 1755 | 494.36 | 347.52 |
|  | 8 | 7625.85 (649.41) | 1013.52 (35.94) | 7.51 (0.47) | 1877.5 | 528.87 | 371.78 |
|  | 9 | 1957.52 (96.7) | 351.46 (7.54) | 5.57 (0.31) | 1392.5 | 392.25 | 275.74 |
|  | 10 | 2006.05 (592.53) | 550 (0) | 3.64 (1.07) | 910 | 256.34 | 180.19 |
|  | 11 | 3599.47 (376.53) | 599.58 (19.83) | 6.01 (1.38) | 1502.5 | 423.24 | 297.52 |
|  | 12 | 3531 (17.39) | 379.10 (10.51) | 9.32 (0.25) | 2330 | 656.34 | 461.38 |
|  | 13 | 5659.1 (234.21) | 908.57 (10.48) | 5.77 (0.29) | 1442.5 | 406.34 | 285.64 |
|  | 14 | 5580.32 (1028.29) | 606.37 (14.68) | 9.18 (1.56) | 2295 | 646.47 | 454.45 |
|  | 15 | 1107.29 (125.06) | 138.52 (15.53) | 7.99 (0.1) | 1997.5 | 562.67 | 395.54 |
|  | 16 | 2525.53 (340.44) | 331.98 (39.48) | 7.63 (0.96) | 1907.5 | 537.32 | 377.72 |
|  | 17 | 5765.19 (841.25) | 1027.33 (5.77) | 5.61 (0.82) | 1402.5 | 395.07 | 277.72 |
|  | 18 | 3238.51 (749.09) | 384.49 (89.85) | 8.43 (0.45) | 2107.5 | 593.66 | 417.32 |
|  | 19 | 1309.62 (222.08) | 272.28 (9.71) | 4.82 (0.87) | 1205 | 339.43 | 238.61 |
|  | 20 | 2271.04 (0.09) | 395 (0) | 5.74 (0) | 1435 | 404.22 | 284.16 |
| Total  Mean (SD) |  | 3944.63 (2675.93) | 607.43 (399.02) | 6.55 (1.57) | 1637.5 | 461.26 | 324.25 |

Notes: The cost of providing lunch each day was recorded by the catering team in 38 schools (20 control and 18 intervention). The other two intervention schools did not have a lunch provision during the intervention year. For analysis, the overall average lunch cost per year for the intervention group was assumed for the two intervention schools which did not have lunch provision during the intervention year.

***** Mean cost per day was measured by taking the average of the total daily cost of lunch divided by the number of diners on each given day. ******Average cost of lunch per diner per day was multiplied by the number of days (250) when schools were open during the 12 month trial period to obtain estimated annual lunch cost per child.

**Table G** Parents/main carers’ workshop attendance time cost

|  | Workshop | Average cost of workshop time for each family member in employment ^A^ | Average cost of workshop time for each family member not in employment ^B^ | Number of paid family members attending the workshop ^C^ | Number of unpaid family members attending the workshop ^D^ | Average cost of each family member time ^E^ |
| --- | --- | --- | --- | --- | --- | --- |
| Component 1  (main carers) | Education | 65 * 2 | 18.3 * 2 | 61% * 683 | 39% * 683 | 93.48 Yuan (18.6 £) |
|  | Family quiz | 65 * 1 | 18.3 * 1 | 61% * 491 | 39% * 491 | 46.74 Yuan (9.3 £) |
| Component 3  (parents) | Family game | 65 * 1 | 18.3 * 1 | 61% * 680 | 39% * 680 | 46.74 Yuan (9.3 £) |
| Total | | | | | | 186.96 Yuan  (37.2 £/52.66 $) |

Notes:

**^E^ =** Average cost of each family member time = (A*C) + (B*D) / total attendees based on the process evaluation data

**^A^** = Average cost of workshop time for each family member in employment = average hourly wage rate * workshop hours over trial duration

**^B^** = Average cost of workshop time for each family member not in employment = average hourly leisure rate * workshop hours over trial duration

**^C^** = Number of paid family members attending the workshop = 61% * total attendees based on the process evaluation data

**^D^** = Number of unpaid family members attending the workshop = 39% * total attendees based on the process evaluation data

**Table H** Outcomes at baseline and 12 months

|  | Mean (SD) | | Adjusted Mean (95% CI) | | | |
| --- | --- | --- | --- | --- | --- | --- |
| Outcomes | Control  group | Intervention group | Difference ^a^  (Intervention  vs control) | p-value | Difference ^b^  (Intervention  vs control) | p-value |
| Baseline | | | | | | |
| Chinese tariff | | | | | | |
| CHU-9D utility | 0.919 (0.094) | 0.921  (0.094) |  | | | |
| EQ-5D-3L utility | 0.969 (0.065) | 0.970  (0.067) |  |  |  |  |
| Post-imputation | | | | | | |
| CHU-9D utility | 0.935 (0.066) | 0.940  (0.069) |  | | | |
| EQ-5D-3L utility | 0.962 (0.087) | 0.964  (0.082) |  |  |  |  |
| At 12 months | | | | | | |
| Chinese tariff | | | | | | |
| CHU-9D QALYs | 0.916 (0.072) | 0.923  (0.069) | 0.006  (0.000 to 0.011) | 0.044 | 0.006  (0.000 to 0.012) | 0.046 |
| EQ-5D-3L QALYs | 0.972 (0.047) | 0.973  (0.052) | 0.002  (-0.002 to 0.005) | 0.363 | 0.001  (-0.001 to 0.004) | 0.450 |
| Post-imputation | | | | | | |
| CHU-9D QALYs | 0.933 (0.052) | 0.938  (0.046) | 0.004  (0.000 to 0.006) | 0.041 | 0.003  (-0.000 to 0.007) | 0.059 |
| EQ-5D-3L QALYs | 0.966 (0.063) | 0.968  (0.066) | 0.001  (-0.002 to 0.004) | 0.337 | 0.002  (-0.002 to 0.008) | 0.442 |

Notes:

CHU-9D, Child Health Utility 9D; CI, Confidence Interval; EQ-5D-3L, Euro-QoL instrument; QALYs, Quality-Adjusted Life Years; SD, Standard Deviation

**^a^** = baseline adjusted model: adjusted for school clustering and baseline outcome.

**^b^** = further adjusted model: adjusted for baseline outcome, prespecified school-level (i.e., whether the school provides midmorning snack, whether the school has an indoor activity room) and child-level sociodemographic (i.e., age, sex, and mother education level) and behavioural (daily average servings of fruit and vegetables, weekly servings of unhealthy snacks and sugar-added drink, objectively measured time in MVPA [minutes/24 hours] and objectively measured sedentary time [minutes/24 hours]) covariates.
